# Artificial intelligence in oral health surveillance among under-served communities

**DOI:** 10.6026/973206300191329

**Published:** 2023-12-31

**Authors:** Anushree Tiwari, Anirbhan Ghosh, Pankaj Kumar Agrawal, Arjun Reddy, Deepika Singla, Dhaval Niranjan Mehta, Gaurav Girdhar, Kapil Paiwal

**Affiliations:** 1 Clinical Quality and Value, American Academy of Orthopaedic Surgeons, Rosemont, USA; 2 Department of Orthodontics and Dentofacial Orthopedics, Bhabha College of Dental Sciences, Bhopal, M.P., India; 3 Department of Oral Pathology and Microbiology, Maitri College of Dentistry and Research Centre, Anjora, Durg, Chhattisgarh, India; 4 Manipal College of Dental Sciences, Manipal, India; 5 Department of Conservative Dentistry and Endodontics, Desh Bhagat Dental College and Hospital, Malout, India; 6 Department of Oral Medicine and Radiology, Narsinbhai Patel Dental College and Hospital, Sankalchand Patel University, Visnagar, Gujarat, India; 7 Department of Periodontology, Karnavati School of Dentistry Karnavati University, Gandhinagar, Gujarat, India; 8 Department of Oral and Maxillofacial Pathology, Daswani Dental College and Research Center, Kota, Rajasthan, India

**Keywords:** AI, oral surveillance, underserved communities

## Abstract

A sizable percentage of the population in India still does not have easy access to dental facilities. Therefore, it is of interest to document the role of artificial intelligence (AI) in oral surveillance of underserved communities. Available data shows that AI makes it possible to screen, diagnose, track, prioritize, and monitor dental patients remotely via smart devices. As a result, dentists won't have to deal with simple situations that only require standard treatments; freeing them up to focus on more complicated cases. Additionally, this would allow dentists to reach a broader, more underprivileged population in difficult-to-reach places. AI fracture recognition and categorization performance has shown promise in preliminary testing. Methods for detecting aberrations are frequently employed in public health practise and research continues to be focused on them.

## Background:

One of the most underserved domains of healthcare is dental care. The most common dental ailments like dental caries, including periodontal disorders, are the main causes of visits to a dental office [1-2]. Considering having a high incidence, a significant portion of the populace still lacks convenient access to dental facilities [3-4]. The strain on the world economy is increased by oral problems, either directly or indirectly. Dental problems indirectly cause absences from work. When someone has a dental problem or an oral problem, they frequently visit a dental office or hospital for care [5- 6]. This is referred to as the dentistry services clinic-based paradigm. In the years that follow, this well-established paradigm will experience considerable modifications [7- 8]. Dental treatments will be offered distantly and actively using telecommunication-based systems and information technology (IT) or dental services will be delivered to patients' homes using mobile dental vehicles [9-10]. It can be very beneficial to shift the focus of treatment from therapeutic to preventative by distant oral surveillance. Comprehensive population screenings could lead to a dramatic decline in oral health issues [11-12]. Most common dental problems, including caries, can be prevented at the first sign of demineralization if they are caught in time [13- 14]. Distant oral monitoring can also be beneficial in screening for more fatal diseases like mouth cancer in order to decrease the number of deaths and disabilities associated with malignant lesions and premalignant conditions by early diagnosis [15-16]. In addition, oral health surveillance can also benefit the inmates in prison as they also include vulnerable group of people who are devoid of oral health facilities and found to have higher prevalence of dental decay and compromised periodontal statuses [17- 18]. For a dental professional, radiographs are a crucial diagnostic tool that can be quickly shared over the internet for tele-consultations. Digital radiography has made it practicable to accurately determine an object's dimension, angles, location, and density [19-20]. Each of the many pixels that make up a digital radiograph produces light with a distinct brightness. In accordance with the variation in radiopacity, cognitive applications and programmes can read and decipher radiographs [21- 22]. The programme may indicate the existence of caries of teeth, variations in bone levels due to periodontal illnesses, and the implementation of cephalo-metric assessment during diagnostic phase of orthodontic treatment [23-24]. These findings can be verified by clinician through remote oral surveillance using AI. The AI algorithm can even recommend a few methods of therapy for timing and procedure of restoration of the carious teeth because AI-based programmes are able to determine the extensiveness of the lesion [25- 26]. AI makes it possible to screen, diagnose, track, prioritize, and monitor dental patients more effectively remotely via smart devices. As a result, dentists won't have to deal with simple situations that only require standard treatments; freeing them up to focus on more complicated ones [27-28]. Additionally, this would allow dentists to reach a broader, more underprivileged population in difficult-to-reach places. Therefore, it is of interest to document the role of AI in oral surveillance among underserved communities like in India.

## Methods and Materials:

## Search Strategy:

The review was prepared in accordance with the Preferred Reporting Items for Systematic Reviews and Meta-Analyses (PRISMA) guidelines. Before being designated, reviewers received training in the two stages of eligibility testing for inclusion criteria and screening of abstracts and full-text evaluation was done. The screening was completed using the Rayyan software. While one observer read across all the outcomes, the other the remaining three researchers independently screened 33.33% of the total search findings twice. After reviewing the abstracts, the panel of reviewers met to resolve their differences and created a final list of full text screening. Two unbiased manual ratings assessed the full-text papers with respect to inclusion criteria. The scientific group's evaluators and panel heads came to a consensus on the final list of articles that would be taken into consideration.

## Inclusion criteria and exclusion criteria:

The following genres of AI-related original research, literature reviews, systematic reviews, scientific communications, letter to editors, and various other preprints were acceptable for inclusion: (1) AI in oral surveillance in underserved communities (2) AI applied to screen, diagnose, track, prioritize, and monitor dental patients remotely via smart devices; (3) AI in remote oral surveillance of dental caries, oral cancer, oral pre-cancer, (4) AI in remote oral surveillance of radiographs. The following criteria were listed as exclusion criteria: (1) Non-English documents were excluded;(2) Works that are published by non-academic outlets (such blogs, newspapers, and magazines).

## Data extraction:

After complete reading of all the selected studies, two researchers extracted following details from each study. These were: details of authors with the year of publication of manuscript. They then obtained details about the main disease for which application of AI was sought. That may be diagnosis of dental caries, diagnosis of head and neck cancer, bone age, injury surveillance etc. There was extraction of details regarding type of data utilised like medical imaging, genomics, demographic details etc. Details were also extracted regarding outcome of the study and AI model incorporated in the study.

The effectiveness of the chosen studies was evaluated using the "risk of bias" technique developed by the Cochrane Collaboration. Each of the seven bias risk domains-random sequence generation, allocation concealment, participant and staff blinding, blinding of outcome assessment, incomplete outcome data, selective reporting, and other bias was subjected to an individual critical examination. Each domain was categorized as having a low, unclear, or high risk of bias. Two independent researchers extracted the qualitative and quantitative data, evaluated the risk of bias, and extracted the information. Discussions amongst the evaluators were used to settle disagreements.

## Results:

**Figure 1:**
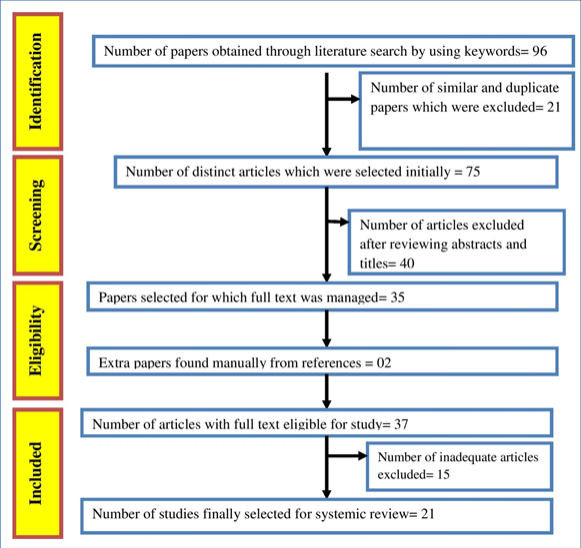
PRISMA flowchart showing selection of studies

The number of papers obtained through literature search by using search terms was ninety-six. The number of similar and duplicate papers that were excluded was twenty-one. 75 distinct articles were selected initially. Forty articles excluded after reviewing abstracts and titles. Full text was managed for 35 articles. Two extra papers found manually from references. The number of articles with full text available for study was 37. In the final assessment 15 inadequate articles were excluded. Finally, twenty-two articles were selected for this review (Figure 1).

## Characteristics of Manuscripts:

The manuscripts covered different aspects of oral as well as head and neck surveillance in underserved communities through artificial intelligence. It covered different aspects like dental caries, periodontal status, head and neck cancer, radiology, fracture, and injuries, COVID 19, health and social care, public health surveillance, infections, musculoskeletal pain etc. The models of AI used were R (reinforcement), ML (machine learning), DL (Deep learning) and RL (Reinforcement learning). The challenges and opportunities of each publication were also evaluated (Table 1).

## AI in dental caries and periodontal problems:

A study [4] provided details of AI in Dental Caries Diagnosis involving medical imaging. They used ML model of AI for classification and segmentation. Challenges were that the inferences that can be drawn regarding the accuracy of a neural network's ability for determining the presence of caries are complicated by variations in outcomes of different studies. Additionally, a comparison of neural network and dental findings is required. This study used a variety of different neural networks and result measurements. A study [5] used ML mode of AI for prediction of head and neck cancer using genomic data. Prognostic forecasting of HNC can benefit from the use of ML approaches for the study of genetic data. Use of AI in a clinical situation may be facilitated by the incorporation of more extensive, more diverse, and diverse information sets, more precise validation results, and a variety of categorization and selecting features strategies. Investigators [6] demonstrated R, ML, DL mode of AI in surveillance of oesophageal cancer. Distinct risk categories of patients sorted by ML models show a considerable or marginally significant variation in survival rates. The ML approach has recently made strides, and its prospects for the future show that it has the ability to offer new quantifiable imaging indicators in medical radiology. Prospective big multi-center research with standardised procedures for imaging and coordination amongst many centres are advised to increase the applicability of ML techniques. Some studies demonstrated the application of ML mode of AI in remote surveillance for cancer. There is no research projects on ML were found to utilize a tool named Comprehensive Geriatric Assessment, a crucial technique for enhancing the care of older cancer patients.

## AI in musculo-skeletal pain, infections and bone age assessment:

A study [9] showed the application of R, ML in surveillance of bone age using medical imaging data in diagnose -classification (bone age of a subject). Studies took into account socioeconomic inequalities and additional (regions of interest) ROIs besides the hand and wrist. High variability in the research makes comparison difficult, as there were a few investigations that took ethnic variations into account and no research that took socioeconomic factors into account. A study [11] demonstrated use of ML in injury surveillance using textual injury data. It is likely that we are going to keep seeing development and advances in our understanding of AI based text mining in the head and neck injury-prevention field due to improvements in methodologies for data mining, greater ability for evaluation of big databases, participation of computer researchers in the avoidance of injuries area, as well as more thorough application and explanation of verification approaches in text mining methodologies. The machine learning methodologies have some drawbacks, including (1) issues with universality of results, (2) issues with source data, (3) difficulties applying complicated models, and (4) restrictions in integrating domain as well as data mining expertise. A study [12] represented application of ML in incident reporting and adverse event analysis. In the specific area of categorization of reports of unfortunate and unfavourable occurrences, NLP can produce beneficial knowledge from unstructured data. In adverse incident evaluation, it's critical to comprehend why or what accidents are happening. A study [13] demonstrated application of ML in musculo-skeletalpain. MVPA (average to strenuous physical activity) assessments based on structural or operational magnetic resonance imaging may be able to distinguish between people with illness and normal controls as well as between noxious as well as non-noxious stimuli, according to initial and evolving evidence. Findings of a study [14] demonstrated application of R, ML models of AI in surveillance of infections using demographics, environmental data, EHR, clinical data, laboratory test, etc. The use of artificial intelligence for automated identification of transmissible illnesses is well established in the scientific literature. In order to identify technological trends and advancements in the field, AI simulations for disease forecasting with multiple characteristics and algorithms should be reviewed throughout time.

## AI in oral surveillance involving radiology and COVID 19 situations:

A study [15] used R, ML approach of AI for oral surveillance in COVID-19 involving empirical and simulation data. It is concluded that government officials may use AI and ML to design methods for managing the COVID-19 pandemic. The impact of treatments may alter significantly over time due to the national vaccination programme and the potential for new COVID-19 variants to have differing transmission tendencies. A study [17] demonstrated the application of ML mode of AI in detecting and diagnosing in COVID-19 situations. The increased use of data mining and machine learning techniques in the medical industry may create the ideal conditions for progress. Findings demonstrated that investigators must go forward with the knowledge they acquire, concentrate on finding answers for CoV issues, and implement new advancements. A former study [19] demonstrated application of ML in dental and maxillofacial radiology for oral screening in communities living in remote areas and who are underserved. The included research' AI models demonstrated numerous clinical uses in DMFR. Before implementing the AI algorithms in clinical practise, it is still important to further confirm their validity and relevance. A study [20] used ML model of AI for detection and classification of fracture involving medical imaging technique like X-ray, CT. Artificial intelligence-based fracture diagnosis and classification has shown encouraging early results. AI could improve how doctors understand and communicate probabilistic activities, like head and neck surgery. The main barrier to AI incorporation into healthcare processes is the lack of sufficient authoritative projects for training and testing. When there is a lack of certainty, the next stage will be to implement AI to increasingly difficult diagnostic and therapeutic settings. Future research should focus on legislative regulation as well as better assess if it can be implemented in clinical practise. A study was conducted to [22] demonstrate application of ML in radiology for diagnosis purpose. NLP programmes may handle a lot of data via robotics and add additional capabilities to healthcare workflows. The growing number of NLP uses in radiology may be improved by setting goals for performance, report standardisation, and independent verification, even if the effectiveness of NLP platforms tends to be high and only a few applications are truly employed in everyday clinical practise or study.

## Outcomes of risk of bias assessment:

There were some studies [5,10,11, 14,15,17] that were found to have maximum risk of bias. On the other hand there were some research [6, 7,9,16, 18,19,20, 22,24,25] having moderate risk of bias. Similarly, there were studies [4,12, 13,23] that were found to have minimum risk of bias. Details of analysis of risk of bias have been given in Table 2.

## Discussion:

By recognizing high-risk groups and carrying out routine distant oral assessments destructive oral diseases can be avoided. By considering the data on high risk factors, practices, socioeconomic status, and genomic information, artificial intelligence (AI) has been utilized to forecast individuals who are at elevated risk of oral cancers, lichen planus and leukoplakia [1]. Oral squamous cell carcinoma has been successfully identified through remote surveillance using straightforward techniques such intraoral photographs. Auto-fluorescence probe for oral cancer based on smartphone has been developed to detect oral malignancies and classify them applying AI in addition to intraoral photography-based remote oral surveillance [2]. Remote discussions will be possible thanks to the installation of these probes at primary dental centres for high-risk groups. Even without the presence of a trained dental professional, these kinds of probes can be used [3].

It can be used for oral surveillance of underserved communities also. Additionally, it would make it possible to lessen the financial impact of oral health disorders [4]. Distant dental work in forensics has greatly benefited from AI. With the aid of models generated by machine learning, full-face rebuilding may be done using lateral cephalo-grams radiographs, and the programme can estimate the age of the individual according to medical photos or radiography [5]. This review is an attempt to present the available literature on the role of AI in oral surveillance of underserved communities. The manuscripts covered different aspects of oral as well as head and neck surveillance in underserved communities through artificial intelligence. It covered different aspects like dental caries, periodontal status, head and neck cancer, radiology, fracture, and injuries, COVID 19, health and social care, public health surveillance, infections, musculoskeletal pain etc. The models of AI used were R, ML, DL and RL. The challenges and opportunities of each publication were also evaluated. A study [4] provided details of AI in Dental Caries Diagnosis involving medical imaging. They used a ML model of AI for classification and segmentation.

Challenges are that the inferences that can be drawn regarding the accuracy of a neural network's ability for determining the presence of caries are complicated by variations in outcomes of different studies. Additionally, a comparison of neural network and dental findings is required. We employed a variety of different neural networks and result measurements. A study [5] used ML mode of AI for prediction of head and neck cancer using genomic data. Prognostic forecasting of HNC can benefit from the use of ML approaches for the study of genetic data. Use of AI in a clinical situation may be facilitated by the incorporation of more extensive, more diverse, and diverse information sets, more precise validation results, and a variety of categorization and selecting features strategies. A study [6] demonstrated R, ML, DL mode of AI in surveillance of oesophageal cancer in patients living in remote areas. Distinct risk categories of patients sorted by ML models show a considerable or marginally significant variation in survival rates. The ML approach has recently made strides, and its prospects for the future show that it has the ability to offer new quantifiable imaging indicators in medical radiology. Prospective big multi-center research with standardised procedures for imaging and coordination amongst many centres are advised to increase the applicability of ML techniques. A study [7] demonstrated application of ML mode of AI in remote surveillance of cancer. The findings highlight limitations that no research projects on ML were found to utilise a tool named Comprehensive Geriatric Assessment, a crucial technique for enhancing the care of older cancer patients. A study [10] evaluated diagnosis of oral squamous cell carcinoma using ML, Dl mode of AI by evaluating clinic-pathologic, imaging or genomic data in remote population. According to observed improvements in predictive accuracy, machine learning has the capacity to enhance prediction of outcomes in kidney transplant and support decision-making in medicine. Future studies should concentrate on employing machine learning techniques radiographs are an essential diagnostic tool for dentists that can be swiftly shared online for tele-consultations. It is now possible to precisely determine an object's size, angles, location, and density thanks to advances in digital radiography [11-12]. A digital radiograph's many pixels each create light with a unique brightness. Cognitive apps and software can interpret and decipher radiographs based on the variation in radiopacity [13-14]. The programme may reveal the presence of dental caries, alterations in bone levels brought on by periodontal diseases, and the use of cephalo-metric assessment during the orthodontic treatment's diagnostic phase [15-16].

These results can be independently validated by a doctor using AI-powered remote oral surveillance. Since AI-based programmes can assess the severity of the lesion, they can even suggest a few therapeutic approaches for the time and method of restorative dentistry for carious teeth [17-18]. A study [19] demonstrated application of ML in dental and maxillofacial radiology for oral screening in communities living in remote areas and who are underserved. AI models demonstrated numerous clinical uses in DMFR. Before implementing the AI algorithms in clinical practise, it is still important to further confirm their validity and relevance. A study [20] used ML model of AI for detection and classification of fracture involving medical imaging technique like X-ray and CT. Artificial intelligence-based fracture diagnosis and classification has shown encouraging early results. AI could improve how doctors understand and communicate probabilistic activities, like head and neck surgery. The main barrier to AI incorporation into healthcare processes is the lack of sufficient authoritative projects for training and testing. When there is a lack of certainty, the next stage will be to implement AI to increasingly difficult diagnostic and therapeutic settings. Future research should focus on legislative regulation as well as better assess if it can be implemented in clinical practise.

## Conclusion:

AI makes it possible to screen, diagnose, track, prioritize, and monitor dental patients more effectively remotely via smart devices. As a result, dentists won't have to deal with simple situations that only require standard treatments; freeing them up to focus on more complicated ones. Additionally, this would allow dentists to reach a broader, more under-privileged population in difficult-to-reach places. AI fracture recognition and categorization performance has shown promise in preliminary testing. Methods for detecting aberrations are frequently employed in public health practise and research continues to be focused on them. There is growing proof that cutting-edge techniques, such as machine learning can perform better than time-series. The data gathered and assessed will be crucial for determining the order in which new methods for oral surveillance of marginalised areas should be developed.

## Figures and Tables

**Table 1:** Important features of studies included in the study

**Author**	**Health/Disease**	**Data type**	**Outcome**	**AI Modelling technique: R, ML, DL, RL**	**Challenges**	**Opportunities**
[11]	Injury surveillance	Textual injury data	Screening	ML	Difficulties in applying complicated models,	There is development and advances in AI based text mining in the head and neck injury-prevention field due to improvements in methodologies for data mining,
[24]	Improve case detection	EMR (electronic medical records)	Detection	ML	More standardised research is required.	Content in EMRs can be retrieved using freely available data gathering tools
[22]	Radiology	EHR and others	Diagnosis	ML	Few applications are employed in everyday clinical practise or study..	NLP (neural learning processing) programmes may handle a lot of data via robotics and add additional capabilities to healthcare workflows.
[13]	Musculoskeletal pain	Medical Image (MRI and fMRI)	Classification	ML	Combining behavioural, genetic, and phenotypic information into analysis to create delicate and precise markers	MVPA assessments based on structural or operational magnetic resonance imaging may be able to distinguish between people with illness and normal controls
[21]	Capture clinical information	Clinical notes, radiology reports, pathology reports, biomedical literature	Capturing and standardizing un structures clinical information	ML		The data gathered and assessed here shall prove crucial for determining the creation of new strategies for medical NLP.
[5]	Head and neck cancer(HNC)	Genomic data	Prediction	ML	Iin corporation of more extensive, information sets required	The prognostic forecasting of HNC can benefit from the use of ML approaches for the study of genetic data.
[8]	Graft failure	Living and deceased donor transplants data (10). Deceased donor transplant (6). Living donor transplant information (1)	Prediction	ML	Future research should focus on modelling time-to-event information	ML has the capacity to enhance prediction of outcomes in kidney transplant and support decision-making in medicine.
[9]	Bone Age	Medical Image (Xray)	Diagnose -Classification (Bone age of a subject)	R, ML	High variability in the research	Studies took into account socioeconomic inequalities and additional (regions of interest) ROIs besides the hand and wrist.
[20]	Fracture	Medical Image (Xray, CT)	Detection and classification	ML	Lack of sufficient authoritative projects for training and testing.	AI could improve how doctors understand and communicate probabilistic activities, like head and neck surgery..
[16]	Critical care	EHR, vital signs, registries	Prediction	ML	These AI algorithms demand enormous databases for training,	In each of the three domains, artificial intelligence for CDS is being used more and more.
[12]	Incident reporting and adverse event analysis	IRS (Incident Reporting System), EHR, and others	Classification	ML	NLP require bigger databases,	, NLP can produce beneficial knowledge from unstructured data.
[25]	Public health surveillance	HER (health electronic records)	Detection	R, ML	These methods have not yet been widely used in practise.	Methods for detecting aberrations are frequently employed in public health practise.
[4]	Dental Caries Diagnosis	Medical image	Classification and segmentation	ML	neural network's ability for determining the presence of caries are complicated by variations in outcomes of different studies.	This review's studies employed a variety of different neural networks and result measurements.
[7]	Cancer	Demographic, Clinical and Histopathological data	Prediction	ML	The data highlight limitations in existing research.	
[14]	Infections	Demographic, environmental data, EHR, clinical data, laboratory test, etc…	Prediction	R, ML	AI algorithms should be reviewed throughout time.	The use of artificial intelligence for automated identification of transmissible illnesses is well established in the scientific literature.
[17]	COVID-19	Patients	Detecting and diagnosing	ML	Investigators concentrate on finding answers for CoV issues, and implement new advancements.	The increased use of data mining and machine learning techniques in the medical industry may create the ideal conditions for progress.
[18]	COVID-19	Medical Images (CT, X-ray)	Detection and classification	ML, DL	Lack of evaluation and testing of AI classification algorithms in evaluation of COVID-19 medical images.	The assessment and testing of COVID-19 AI categorization approaches is seen as a multi-complex parameter issue.
[19]	Dental and maxillofacial radiology (DMFR)	Medical Image (X-ray)	Screening	ML	It is important to further confirm their validity and relevance.	The included research' AI models demonstrated numerous clinical uses in DMFR.
[23]	Health and social care	Clinical data	Not available	ML	The inadequate and inconsistent evidence of AI's potential to enhance taking decisions	it has assisted in identifying how the scientific proof base already in place should be conceptualized in this field moving forward
[6]	Esophageal cancer	Radiomics	Classification	R, ML, DL	ML models show a considerable or marginally significant variation in survival rates.	ML has the ability to offer new quantifiable imaging indicators in medical radiology.
[10]	Oral squamous cell carcinoma	Clinopathologic, imaging or genomic data.	Diagnosis	ML, DL	Additionally, laws and regulations are required.	In research on oral cancer, machine learning AI algorithms have demonstrated potential diagnostic as well as prognostic capabilities.
[15]	COVID-19	Empirical and simulation data	Prediction	R, ML	Potential for new COVID-19 variants to have differing transmission tendencies.	Government officials may use AI and ML to design methods for managing the COVID-19 pandemic..
Footnotes: AI (Artificial intelligence), R (reinforcement), ML (machine learning) ,DL (Deep learning) ,RL (Reinforcement learning)

**Table 2:** Summary Cochrane ROB assessment for individual studies

**Reference**	**Sequence generation**	**Allocation concealment**	**Blinding of participants, personnel**	**Blinding of outcome assessors**	**Incomplete Outcome data**	**Selective outcome reporting**	**Other Sources of bias**	**Overall risk of bias**
[11]	+	-	+	+	+	+	+	-
[24]	+	?	?	?	+	+	+	?
[22]	+	?	?	?	+	+	+	?
[13]	+	+	+	+	+	+	+	+
[21]	?	?	?	?	+	+	+	?
[5]	+	-	+	+	+	+	+	-
[8]	+	-	+	+	+	+	+	-
[9]	+	?	?	?	+	+	+	?
[20]	+	?	?	?	+	+	+	?
[16]	+	?	?	?	+	+	+	?
[12]	+	+	+	+	+	+	+	+
[25]	+	?	?	?	+	+	+	?
[4]	+	+	+	+	+	+	+	+
[7]	?	?	?	?	+	+	+	?
[14]	+	-	+	+	+	+	+	-
[17]	+	-	+	+	+	+	+	-
[18]	+	?	?	?	+	+	+	?
[19]	+	?	?	?	+	+	+	?
[23]	+	+	+	+	+	+	+	+
[6]	?	?	?	?	+	+	+	?
[10]	+	-	+	+	+	+	+	-
[15]	+	-	+	+	+	+	+	-
Minimum Risk of Bias represented by +; Moderate Risk of Bias represented by?; Maximum Risk of Bias -
